# CDX2 enhances natural killer cell–mediated immunotherapy against head and neck squamous cell carcinoma through up‐regulating CXCL14

**DOI:** 10.1111/jcmm.16253

**Published:** 2021-03-17

**Authors:** Haitao Wang, Shanji Nan, Ying Wang, Chengbi Xu

**Affiliations:** ^1^ Department of Otolaryngology Head and Neck Surgery Jilin University Second Hospital Changchun China; ^2^ Department of Neurology Jilin University Second Hospital Changchun China; ^3^ Department of Gastroenterology Jilin University First Hospital Changchun China

**Keywords:** CDX2, CXCL14, HNSCC, immunotherapy, NK cells

## Abstract

(NK) cells are at the first line of defence against tumours, but their anti‐tumour mechanisms are not fully understood. We aimed to investigate the mechanism by which NK cells can mediate immunotherapy against head and neck squamous cell carcinoma (HNSCC). We collected fifty‐two pairs of HNSCC tissues and corresponding adjacent normal tissues; analysis by RT‐qPCR showed underexpression of CXCL14 in HNSCC tissues. Primary NK cells were then isolated from the peripheral blood of HNSCC patients and healthy donors. CXCL14 was found to be consistently under‐expressed in the primary NK cells from the HNSCC patients. However, CXCL14 expression was increased in IL2‐activated primary NK cells and NK‐92 cells. We next evaluated NK cell migration, IFN‐γ and TNF‐α expression, cytotoxicity and infiltration in response to CXCL14 overexpression or knockdown using gain‐ and loss‐of‐function approach. The results exhibited that CXCL14 overexpression promoted NK cell migration, cytotoxicity and infiltration. Subsequent in vivo experiments revealed that CXCL14 suppressed the growth of HNSCC cells via activation of NK cells. ChIP was applied to study the enrichment of H3K27ac, p300, H3K4me1 and CDX2 in the enhancer region of CXCL14, which showed that CDX2/p300 activated the enhancer of CXCL14 to up‐regulate its expression. Rescue experiments demonstrated that CDX2 stimulated NK cell migration, cytotoxicity and infiltration through up‐regulating CXCL14. In vivo data further revealed that CDX2 suppressed tumorigenicity of HNSCC cells through enhancement of CXCL14. To conclude, CDX2 promotes CXCL14 expression by activating its enhancer, which promotes NK cell–mediated immunotherapy against HNSCC.

## 1 INTRODUCTION

Head and neck squamous cell carcinoma (HNSCC), which usually arises from the mouth, nose and throat, with metastasis to neighbouring lymph nodes, is the 6th most common cancer in the world.^1^, ^2^ Besides, smoking and alcohol consumption are the major risk factors to cause HNSCC, and additionally, human papillomavirus (HPV) infection and TP53 mutation are also associated with HNSCC.^3^ Every year, around 500 000 cases are newly diagnosed with HNSCC globally, with approximately 40 000 new cases and consequent 7890 deaths occurring annually in the United States.^4^ Surgery, radiation and chemotherapy, along or in combination with targeted therapy, are used for HNSCC therapy. Given the cytotoxicity of conventional treatment and their failure in the treatment of recurrent and/or metastatic stages of HNSCC, cell‐based immunotherapies are being explored as promising strategies for the treatment of HNSCC.^5^

Cell‐based immunotherapy refers to a treatment strategy that uses the immune system of patients to fight against infections and other diseases such as cancer through a targeted activation or repression of their white blood cells and lymphatic system.^6^ In this scenario, immune mediators including natural killer (NK) cells, dendritic cells (DC), macrophages and cytotoxic T lymphocytes (CTLs) protect the host and eliminate tumours by targeting surface antigens of tumour cells.^7^ Natural killer (NK) cells are so‐named due to their innate and cytotoxic characteristics, which were initially identified as a population of spleen‐derived cytotoxic lymphocytes that were neither B nor T cells.^8^ The NK cells function as the first line of defence against pathogens and tumours through their effects on death‐receptor pathways and granule exocytosis, which are similar to those of CTLs.^9^ The chemokine (C‐X‐C motif) ligand 14 (CXCL14), also known as BRAK, is expressed in a wide range of normal cells, but is especially abundant in epithelial cells. CXCL14 is also expressed in immune cells, where it is involved in immune surveillance by recruiting NK, dendritic cells and T cells.^10^, ^11^ Cicchini et al^12^ uncovered that CXCL14 restoration could significantly inhibit the development of head and neck cancer (HNC) in human papillomavirus (HPV)–positive HNC cells in vivo. However, current knowledge regarding the potential function of CXCL14 in immunotherapy is limited only to the recruitment of NK and/or T cells to the tumour microenvironment, whereas the mechanism by which CXCL14 suppresses tumour development remains unclear.

In this paper, we investigated the underlying molecular mechanism whereby CXCL14 mediates NK cells to target HNSCC. Our research findings reveal that caudal type homeobox 2 (CDX2), upstream regulator of CXCL14, activates CXCL14 enhancer to up‐regulate its expression and enhance the therapeutic efficacy of immunotherapy against HNSCC by NK cells. Furthermore, the CDX2 pathway also provides a potential immunotherapeutic target to improve the treatment of advanced stages of HNSCC.

## 2 MATERIALS AND METHODS

### 2.1 Clinical samples

Head and neck squamous cell carcinoma tissue samples and corresponding adjacent normal tissue samples used in this study were collected from 52 patients undergoing surgical operations at The Second Hospital of Jilin University (March 2016 to March 2018). None of these patients had received radiation therapy or chemotherapy before the operations. Isolated tissue samples were immediately examined by the department of pathology. All samples were frozen in liquid nitrogen before RNA extraction. Based on the TNM classification of malignant tumours of the Union for International Cancer Control, we identified tumour stage and determined histological grade according to Broder's classification system. Meanwhile, 10 mL samples of venous blood sample were collected from 10 healthy volunteers and 10 HNSCC patients for biochemical analysis. The protocols involved in this paper were approved by the Ethics Committee of Jilin University Second Hospital. All procedures in this study involving humans were in accordance with the *Declaration of Helsinki*. Before sample collection, all participants provided written informed consents for entry in the research project.

### 2.2 Natural killer cell isolation and culture

Peripheral blood mononuclear cells (PBMCs) from 10 healthy volunteers and 10 HNSCC patients were isolated according to the manufacturer's protocol of the Ficoll‐Paque Premium kit (GE healthcare; VWR), and NK cells were isolated using a Miltenyi NK cell isolation kit (LS) (Miltenyi Biotech) following manufacturers’ procedures. Purity of the NK cells exceeded 85% and contained less than 1% CD3^+^ T cells. Isolated NK cells were cultured in a human NK cell culture medium with R10 + 5% human AB serum (Sigma).

The NK92 cell line was purchased from cell bank of China Academy of Sciences and was grown in a Minimum Essential Medium (MEM) α medium (Thermo Fisher Scientific) supplemented with 10% foetal bovine serum (FBS) (Thermo Fisher Scientific), 1.5 g/L NaHCO_3_, 2 mM L‐glutamine, 0.2 mM inositol, 0.02 mM folic acid, 0.1 mM 2‐mercaptoethanol and 100 U/ml recombinant human interleukin‐2 (IL‐2). Cells were cultured in a 37℃ incubator under standard conditions with 5% CO_2_.

### 2.3 Lentiviral infection

Gene silencing and overexpression constructs were made based on pSIH1‐H1‐copGFP and pLV‐EGFP‐N vectors, respectively. Vectors used here were purchased from GenePharma Co., Ltd., including oe‐CXCL14 (pLV‐EGFP‐N‐CXCL14), oe‐CDX2 (pLV‐EGFP‐N‐CDX2), sh‐CXCL14 (pSIH1‐H1‐copGF‐CXCL14), sh‐p300 (pSIH1‐H1‐copGF‐p300) and sh‐CDX2 (pSIH1‐H1‐copGF‐CDX2). NK cells were infected by incubation in 1 mL medium without FBS and antibiotics mixed with 2 × 10^6^ TU lentivirus and 1 μg Polybrene (Sigma), followed by observation under an inverted fluorescent microscope after incubation for 2‐3 days. After 48 hours of infection, 1 μg/mL puromycin (Sigma) was supplemented to screen the stably transfected clones. After cell screening and culture for several days, the medium was replaced by standard cell culture medium. When cells reached a logarithmic growth phase and 80% confluence, they were collected and subjected to real‐time quantitative polymerase chain reaction (RT‐qPCR) to detect the target gene expression for evaluating transfection efficiency.

### 2.4 RT‐qPCR

Total RNA from cells or tumour tissues was extracted using TRIzol reagent (Invitrogen). Agarose gel electrophoresis was used to examine the integrity and purity of isolated RNA. Reverse transcription was done using a Primescript™ RT Reagent Kit (RRO37A; TaKaRa). qPCR was performed with an ABI 7500 instrument (Applied Biosystems) to amplify targeted genes and internal control sequences. The PCR system was set up for 25 μL volumes, and GAPDH served as internal control. The 2^−ΔΔ^ *^C^* ^t^ method was used to quantify the relative expression of mRNA.^13^ Primers used for qPCR are listed below (Table 1).

**TABLE 1:** Primer lists for RT‐qPCR

Targeted gene	Forward primer (5′‐3′)	Reverse primer (5′‐3′)
CXCL14 (m)	CCAAGATTCGCTATAGCGAC	CCTGCGCTTCTCGTTCCAGG
CXCL14 (h)	TCCGGTCAGCATGAGGCTCC	CACCCTATTCTTCGTAGACC
CDX2 (m)	ACGCTGCGAGAATCCTCAGAAG	CTGGCAGGAAGAGTCGGAAT
CDX2 (h)	CCACGCTGGGGCTCTCT	GTCTAGCAGAGTCCACGCTC
p300 (m)	GCCAAGTATGCCAACCCTAA	TGTTCATTTGCTGAGCTTGG
p300 (h)	AAACCCACCAGATGAGGA	TATGCACTAGATGGCTCCGCAG
GAPDH **(**m**)**	GGGAAGCCCATCACCATCTT	GCCTTCTCCATGGTGGTGAA
GAPDH **(**h**)**	AGAAGGCTGGGGCTCATTTG	AGGGGCCATCCACAGTCTTC

Abbreviations: h, human; m, mouse.

### 2.5 Western blot assay

Cells were washed twice with prechilled PBS, followed by addition of lysis buffer (R0278; Sigma). Then, cells were centrifuged at 60000 g for 30 minutes at 4℃. The supernatant containing proteins was saved and the protein concentration quantified using a bicinchoninic acid assay (BCA) kit (BCA1; Sigma). 50 μg protein was boiled with 2 × sodium dodecyl sulphate (SDS) loading buffer at 100℃ for 5 minutes. Proteins were separated by 10% SDS‐polyacrylamide gel electrophoresis (PAGE), followed by transferred to a polyvinylidene fluoride (PVDF) membrane. The membrane was blocked with 5% skim milk for 1 hour at room temperature, followed by incubation with diluted primary anti‐CXCL14 (ab137541, 1:1000; Abcam), anti‐natural killer group 2D (NKG2D) (ab203353, 1:500; Abcam), anti‐CDX2 (ab76541, 1:10 000; Abcam), anti‐p300 (ab59240, 1:1000; Abcam) and anti‐glyceraldehyde 3‐phosphate dehydrogenase (GAPDH) (ab181602, 1:10 000; Abcam) antibodies. Next, the membrane was washed three times with Tris‐buffered saline Tween‐20 (TBST) and incubated with horseradish peroxide–conjugated secondary antibody (ab6721, 1:2000; Abcam) for 1 hour at room temperature. After being washed three times with TBST, the membrane was developed using enhanced chemiluminescence (ECL) (BB‐3501; Ameshame) and photographed with a Bio‐Rad imaging system (Bio‐Rad Laboratories). Images were analysed by Quantity One v4.6.2 software by quantifying the grey value of the band with GAPDH as loading control. Experiments were repeated in triplicate.

### 2.6 Immunoprecipitation analysis

Nuclear protein extraction was performed as previously described.^14^ Protein G Dynabeads (Invitrogen) used for Immunoprecipitation (IP) were blocked with 1% bovine serum albumin (BSA) before the experiment. On the day of IP, nuclear proteins were dialysed into the BC200 buffer (20 mM HEPES, pH 7.9, 0.2 mM EDTA, 0.5 mM DTT, 20% glycerol, 0.2% NP‐40 and 200 mM KCl). Then, 5 µg p300 (ab14984; Abcam) or IgG control (ab157532; Abcam) was incubated with Dynabeads for at least 6 hours. 200 µg portions of nuclear proteins were diluted to a concentration of 200 ng/µL with BC200 buffer and precleared with Dynabeads for 2 hours, followed by incubation with Dynabeads‐antibody complex overnight. The beads were washed with BC200 and BC500, two times each. Finally, the beads were resuspended in 30 µL BC200 for subsequent experiments.

### 2.7 Transwell assay

Transwell assay was conducted as previously indicated.^15^ In brief, NK cells were seeded in the upper layer of a Costar 24‐well Transwell plate (8 μm diameter). Next, 600 μL of RPMI 1640 supplemented with 10% FCS and 200 ng/mL IL‐16 (R&D Systems) was placed in the bottom layer of the Transwell plate. After incubation for 4 hours at 37°C, the cell number in each lower well was recorded twice, and the cell migration rate in regular medium was set as 100%. Migration due to chemotaxis was calculated as the percentage of the spontaneous migration.

### 2.8 Enzyme‐linked immunosorbent assay

The supernatant of NK cell cultures was collected and transferred to an Eppendorf (EP) tube, followed by centrifugation at 8000 *g* for 8 minutes. The amount of IFN‐γ and TNF‐α in the supernatant was measured according to the protocol of the Enzyme‐linked immunosorbent assay (ELISA) kit (Abcam).

### 2.9 Cytotoxicity assay

CAL27 (ATCC^®^CRL‐2095) and SCC‐25 (ATCC^®^1628) cell lines were purchased from the American Type Culture Collection (ATCC) and cultured in Dulbecco's modified Eagle medium (DMEM) (Gibco) supplemented with 10% FBS (Invitrogen) in a 37°C incubator with 5% CO_2_. VenorGeM Mycoplasma PCR Detection Kit (Minerva Biolabs) was routinely used to test for mycoplasma contamination. CAL27 and SCC‐25 cells were labelled with 10 µM DDAO‐SE (Molecular Probes; Invitrogen) according to the manufacturer's protocol and incubated at 37°C overnight, followed by the addition of IL‐2‐stimulated human primary NK cells at a ratio of 10:1 and incubation for 4 hours at 37°C. Cells were detached and collected using Trypsin‐EDTA (Invitrogen) and stained with Live/Dead Fixable Green cell stain (Molecular Probes; Invitrogen). The cytotoxicity percentage was quantified by calculating the percentage of dead cells in target cells subtraction of the background from dead cells without effectors.

### 2.10 Tumour spheroid assays

In order to study the infiltration of NK cells, SCC‐25 cell line was used for tumour spheroid assays. SCC‐25 and IL‐2‐stimulated primary NK cells were seeded with a 1:1 ratio into agarose coated 96‐well plates. After 8 hours in culture, individual spheroids (n > 8) were collected, mixed with optimum cutting temperature (OCT) compound (Sakura) and frozen. Tumour spheroids were cryosectioned at a thickness of 7 µm, and the slices were incubated with mouse anti‐human CD45 (clone 2B11 + PD7/26; DAKO). The number of CD45^+^ cells was analysed by a standard two‐step polymer protocol using an Envision kit (DAKO, Germany) and quantified as the percentage of CD45^+^ NK cells of total cell counts of the spheroid cryosections.

### 2.11 Chromatin immunoprecipitation assay

Chromatin immunoprecipitation (ChIP) assay was performed using the EZ‐Magna ChIP kit (EMD Millipore). According to the manufacturer's procedure, NK cells were crosslinked with 4% paraformaldehyde (PFA) for 10 minutes and quenched with glycine. Next, cells were lysed with cell lysis buffer and nuclear lysis buffer and subjected to sonication to fragment chromatin to 200‐300 bp. Sonicated chromatin was then immunoprecipitated with magnetic protein A beads bound with antibodies, namely 2 μg negative control IgG (ab171870; Abcam), or 2 μg H3K27ac (ab203953; Abcam), 2 μg P300 (ab14984; Abcam), 2 μg H3K4me1 (ab8895; Abcam), and 2 μg CDX2 (sc‐134362; Santa Cruz Biotechnology Inc.). Finally, RT‐qPCR was applied to analyse the enrichment of proteins on the enhancer region of CXCL14 (chr5:134906373‐134914969). Primers used for qPCR included forward (5′‐3′): CACCTGCGAAGGAAGGAGTT and reverse (5′‐3′): TGAGGACTTCAGTGGGGTGA.

### 2.12 Tumour‐bearing mouse model

SCC7 is a mouse squamous cell carcinoma cell line that originated from a spontaneously arising HNSCC from C3H/HeN mice. SCC7 cells were cultured in DMEM (Sigma) supplemented with 100 U/mL penicillin, 100 μg/mL streptomycin and 10% FBS. In order to establish the tumour‐bearing mouse model, SCC7 cells were subcutaneously transplanted into syngeneic C3H/HeN mice with a density of 10^6^ cells/50 μL saline/mouse. To eliminate NK cells, mice were intravenously injected with anti‐asialo GM1 antibody before and after experiments on days −2, 0, 2, 4, 6, 13, 20, 27, 34 and 41. Meanwhile, CDX2 and /or CXCL14 overexpression or knockdown lentiviruses (1 × 10^8^ pfu/100 μL) purchased from GenePharma Co., Ltd. were intraperitoneally injected into mice. The diameter of tumours was measured with a digital calliper every 3 days, and the tumour volume was calculated according to the formula as indicated: V = A × B^2^/2, in which V is the tumour volume, A is the maximum tumour diameter, and B is the diameter perpendicular to A.

### 2.13 Immunofluorescence

Mouse tumours were fixed in 4% (w/v) PFA for 3 hours at 4°C, followed by 30% (w/v) sucrose incubation overnight. The next day, samples were embedded in OCT (Tissue‐Tek), frozen on dry ice, cut into 10‐μm‐thick sections with a cryomicrotome and stored at −20°C until staining. Slides were air‐dried for 20 minutes at room temperature and incubated with PBS to remove the OCT. Samples were then incubated in PBS/ 0.2% Triton X‐100 for permeabilization at room temperature for 10 minutes. Next, the samples were blocked with 10% goat serum and 0.1% Triton X‐100 for 1 hour at room temperature, followed by addition of diluted anti‐CD45 primary antibody (ab10558, 1:1000; Abcam) for incubation in staining buffer at 4°C overnight. The next day, samples were washed with staining buffer and then incubated with diluted secondary antibody (ab175475, 1:200; Abcam) incubation in staining buffer for 1 hour in the dark, followed by washing with staining buffer. DAPI was used for counterstaining, and samples were washed with PBS and deionized water. Immunofluorescence (IF) images were captured under an inverted fluorescent microscope (Eclipse Ti), which were quantified by ImageJ (NIH) in at least four randomly selected individual fields of view per sample.

### 2.14 Statistical analysis

All data in this paper were processed using SPSS 21.0 (IBM Corp., Armonk, NY, USA). Measurement data were presented as mean ± standard deviation (mean ± SD). Data from tumour and adjacent normal tissues that were normally distributed were analysed by paired *t* test, while data of other two groups were compared using unpaired *t* test. Data among multiple groups were processed *via* one‐way analysis of variance (ANOVA) and Tukey's post hoc test, and tumour volume data from different time‐points were analysed by repeated measures ANOVA. Statistical difference was presented as *P* < .05.

## 3 RESULTS

### 3.1 CXCL14 expression is down‐regulated in HNSCC and the derived NK cells

**FIGURE 1:**
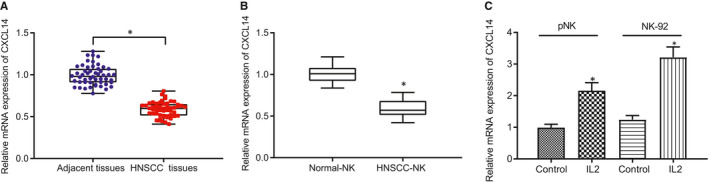
CXCL14 expression is down‐regulated in head and neck squamous cell carcinoma (HNSCC) and the derived NK cells. (A) RT‐qPCR to detect the expression of CXCL14 in clinical samples of HNSCC patients (n = 52). (B) RT‐qPCR to detect the expression of CXCL14 in NK cells from peripheral blood of healthy volunteers (n = 10) and HNSCC patients (n = 10). (C) RT‐qPCR to detect the expression of CXCL14 in IL‐2‐stimulated NK cells and NK‐92 cells. *P < .05 compared with adjacent normal tissues/normal NK/NK‐92 cells. Measurement data were presented as mean ± standard deviation. Data from tumour and adjacent normal tissues were analysed by paired *t* test, while data of two groups were compared using unpaired *t* test. Experiments were repeated 3 times

It has been reported that chemokine CXCL14 expression correlates with the prognosis of colorectal cancer after resection.^16^ Ozawa et al^17^ reported that the anti‐tumour effect of gefitinib in HNSCC was associated with restoration of the gene expression of BRAK/CXCL14. In order to further investigate the role of CXCL4 in HNSCC, we first collected 52 pairs of HNSCC tissue samples and corresponding adjacent normal tissue samples. The expression of CXCL14 mRNA was quantified by RT‐qPCR, which showed that CXCL14 expression was significantly decreased in HNSCC tissues compared with normal tissues (Figure 1A). By analysing the relationship between CXCL14 and clinical characteristics of HNSCC patients, it emerged that CXCL14 expression correlated with the metastasis of HSNCC and TNM classification, without any relation to patient gender and age (Table 2).

**TABLE 2:** The correlation between CXCL14 expression and clinical features of HNSCC patients

Characteristics	Number of cases	CXCL14 expression		*P* value
		Low (n = 26)	High (n = 26)	
Age (year)				
≤60	29	12	16	.266
>60	23	14	10	
Gender				
Female	23	9	13	.262
Male	30	17	13	
Lymph node metastasis				
N0	21	6	15	.011
N1 + N2	31	20	11	
TNM stage				
I + II	22	5	17	<.001
III	30	21	9	

Note

The data were analysed by chi‐square test.

Abbreviations: HNSCC, head and neck squamous cell carcinoma; N0, no regional lymph node metastasis; N1, unilateral cervical and/or unilateral or bilateral retropharyngeal node (s), ≤6 cm in greatest dimension, above supraclavicular fossa; N2, bilateral cervical node (s), ≤6 cm in greatest dimension, above supraclavicular fossa; T1, nasopharynx, oropharynx or nasal cavity; T2, parapharyngeal extension; T3, bony structures and/or paranasal sinuses; TNM, tumour node metastasis.

To investigate whether the function of CXCL14 was correlated with NK cells, we first isolated NK cells from peripheral blood and measured their expression of CXCL14 by RT‐qPCR. The results demonstrated that, compared with the controls, the expression of CXCL14 was notably down‐regulated in NK cells of HNSCC patients (Figure 1B), which suggested that CXCL14 was poorly expressed in the inactivated NK cells. NK cell–mediated anti‐tumour effect has been shown to depend on IL‐2, which is known as a survival factor of NK cells and an enhancer of the cytotoxic properties.^18^ Next, we applied IL‐2 to stimulate the primary NK cells isolated from healthy donors and NK‐92 cells to study the function of CXCL14 on NK cells. RT‐qPCR analysis revealed that CXCL14 expression was considerably increased in IL‐2‐activated primary NK cells and in NK‐92 cells compared with untreated control cells (Figure 1C). Altogether, these data indicated a down‐regulation of CXCL14 in HNSCC and the derived NK cells.

### 3.2 CXCL14 enhances NK cell migration and cytolytic activity

**FIGURE 2:**
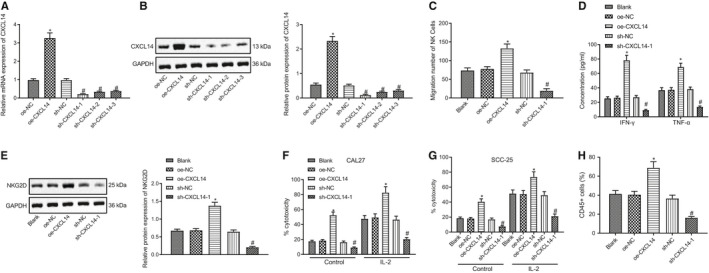
CXCL14 enhances NK cell migration and cytolytic activity. (A) RT‐qPCR to detect CXCL14 expression after silencing or overexpressing CLCX14 by lentivirus. (B) Western blot assay to detect the expression of CLCX14 protein after silencing or overexpressing CLCX14 by lentivirus. (C) Transwell to assess the cell migration. (D) ELISA to measure the levels of IFN‐γ and TNF‐α in the medium of NK cells. (E) Western blot assay to analyse the expression of NKG2D in NK cells. (F) The cytotoxicity analysis of primary NK cells to head and neck squamous cell carcinoma (HNSCC) cell line CAL27. (G) The cytotoxicity analysis of primary NK cells to HNSCC cell line SCC‐25. (H) Immunohistochemistry to examine CD45 expression in SCC‐25 tumour spheroid. *P < .05 compared with oe‐NC group, and ^#^ *P* < .05 compared with sh‐NC group. Measurement data were presented as mean ± standard deviation. Data among multiple groups were processed via one‐way ANOVA and Tukey's post hoc test. Experiments were repeated 3 times

To study whether CXCL14 participates in cancer immunotherapy by NK cells, we first knocked down or overexpressed CXCL14 using lentivirus in NK cells. RT‐qPCR and Western blot results demonstrated that, compared with oe‐NC treatment, CXCL14 expression was significantly increased by oe‐CXCL14. However, in comparison to sh‐NC, the sh‐CXCL14‐1, sh‐CXCL14‐2 and sh‐CXCL14‐3 treatments remarkably down‐regulated the expression of CXCL14 (Figure 2A,B), among which sh‐CXCL14‐1 showed the best knockdown efficacy and was selected for the subsequent experiments. Transwell assay was next applied to test the effect of CXCL14 on the migration of NK cells, which uncovered notably increased migration upon treatment with oe‐CXCL14, but considerably decreased migration by sh‐CXCL14‐1 (Figure 2C). Then, ELISA analysis of the medium from NK cells demonstrated that the levels of IFN‐γ and TNF‐α were elevated by CXCL14 overexpression; however, the levels of these two factors in the medium were remarkably suppressed by CXCL14 silencing (Figure 2D).

NKG2D is an important protein associated with the cytotoxic activity of NK cells.^19^ Therefore, we performed Western blot assay to detect the expression of NKG2D in NK cells. The data revealed that NKG2D expression was considerably increased by oe‐CXCL14 and decreased by sh‐CXCL14‐1 (Figure 2E). Next, we evaluated the cytotoxicity of IL‐2‐stimulated NK cells against HNSCC, CAL27 and SCC‐25 cells, and found that the cytotoxicity of IL‐2‐stimulated NK cells against HNSCC cells was significantly enhanced by oe‐CXCL14, but was reduced by sh‐CXCL14 (Figure 2F,G). Meanwhile, to study the infiltration of NK cells, we conducted tumour spheroid assay using the SCC‐25 cell line. The immunohistochemistry results demonstrated that the number of infiltrated NK cells (CD45^+^ cells) was considerably increased by oe‐CXCL14, whereas their number was remarkably decreased by sh‐CXCL14‐1 (Figure 2H). These data revealed that CXCL4 increased NK cell migration and cytolytic activity.

### 3.3 CXCL14 inhibits tumorigenesis

**FIGURE 3:**
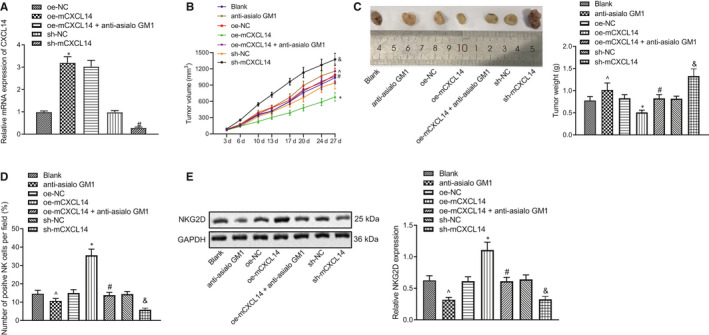
CXCL14 inhibits the growth of head and neck squamous cell carcinoma (HNSCC) in vivo. (A) RT‐qPCR to detect CXCL14 expression in mouse tumours. (B) Measurement of tumour volume. (C) Measurement of tumour weight. (D) IF to detect the NK cell infiltration in mouse tumours. (E) Western blot assay to analyse the expression of NKG2D in mouse tumours. ^*P* < .05 compared with blank group, *P < .05 compared with oe‐NC group, ^#^ *P* < .05 compared with oe‐mCXCL14 group, and & *P* < .05 compared with sh‐NC group. Tumours were excised 3 wks after lentivirus injection, and RT‐qPCR, IF and Western blot assay were performed. Measurement data were presented as mean ± standard deviation. Data among multiple groups were compared via one‐way ANOVA and Tukey's post hoc test, and tumour volume data from different time‐points were analysed by repeated measures ANOVA. Experiments were repeated 3 times

To study the role of CXCL14 in HNSCC and whether this role was associated with effects of NK cells in vivo, we established a tumour‐bearing mouse model using SCC7 cells and then injected CXCL14 overexpression (oe‐mCXCL14) or knockdown (sh‐mCXCL14) lentivirus or corresponding control vectors into the mice. At three weeks after injection, the expression of CXCL14 mRNA in tumours was determined by RT‐qPCR, which showed that CXCL14 expression was significantly up‐regulated in the tumour‐bearing mice injected with oe‐mCXCL14, but notably down‐regulated in those injected with sh‐mCXCL14 (Figure 3A). In addition, it was also found that the tumour volume and weight of mice were considerably decreased upon CXCL14 overexpression, but remarkably increased after CXCL14 knockdown. Meanwhile, the tumour volume and weight reduced by CXCL14 overexpression were significantly increased after treatment with anti‐asialo GM1 (Figure 3B,C).

We then used IF to detect the NK cell infiltration in tumours, which demonstrated that the number of infiltrating CD45^+^ cells in the tumour‐bearing mice was remarkably elevated in response to CXCL14 overexpression, but was decreased in response to CXCL14 knockdown. In the meantime, the number of CD45^+^ cells elevated by CXCL14 was notably decreased after treatment with anti‐asialo GM1 (Figure 3D). Additionally, the NKG2D protein expression was measured by Western blot assay. The results showed that CXCL14 overexpression led to increased NKG2D protein expression, while CXCL14 knockdown caused a reduction in its expression. However, the CXCL14‐induced elevation of NKG2D protein expression was repressed by anti‐asialo GM1 treatment (Figure 3D,E). These results revealed that CXCL14 considerably inhibited the tumour growth in mice which was possibly mediated by NK cells.

### 3.4 CDX2 increases CXCL14 expression *via* activating its enhancer in primary NK cells

**FIGURE 4:**
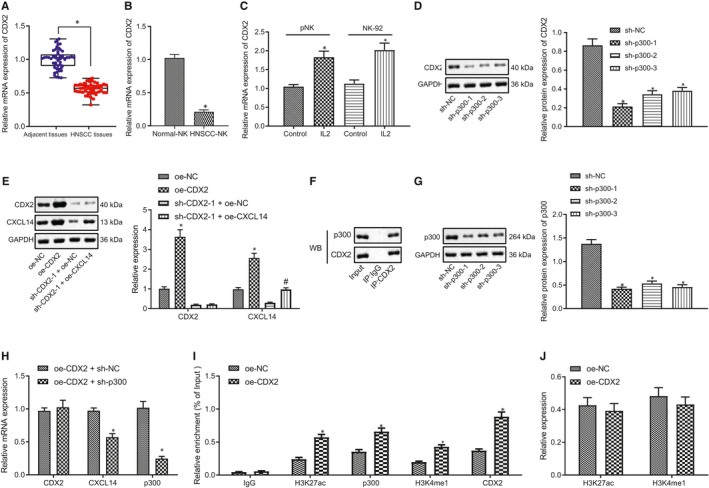
CDX2 increases CXCL14 expression *via* activating its enhancer in primary NK cells. (A) RT‐qPCR to detect CDX2 expression in clinical samples of head and neck squamous cell carcinoma (HNSCC) patients (n = 52). *P < .05 compared with the adjacent normal tissues. (B) RT‐qPCR to detect CDX2 expression in NK cells of peripheral blood of healthy volunteers and HNSCC patients. *P < .05 compared with NK cells from healthy volunteers. (C) RT‐qPCR to detect CDX2 expression in IL‐2‐stimulated primary NK cells and NK‐92 cells. *P < .05 compared with control NK cells and NK‐92 cells. (D) Western blot assay to analyse the expression of CDX2 after silencing CDX22 in primary NK cells by lentivirus. *P < .05 compared with sh‐NC group. (E) RT‐qPCR and Western blot assay to detect the expression of CDX2, CXCL14, and p300 in NK cells. *P < .05 compared with oe‐NC group, and ^#^ *P* < .05 compared with sh‐CDX2‐1 + oe‐NC group. (F) IP assay to analyse the interaction between p300 and CDX2 in primary NK cells. (G) Western blot assay to analyse the expression of p300 after knocking p300 down in primary NK cells. *P < .05 compared with sh‐NC group. (H) RT‐qPCR to detect the expression of CDX2, CXCL14 and p300. *P < .05 compared with oe‐CDX2 + sh‐NC group. (I) ChIP to analyse the effect of CDX2 on the enrichment of H3K27ac, p300 and H3K4me1 in the enhancer of CLCX14. (J) Western blot assay to analyse the expression of H3K27ac and H3K4me1. *P < .05 compared with oe‐NC group. Measurement data were presented as mean ± standard deviation. Data from tumour and adjacent normal tissues that were normal distributed were analysed by paired *t* test, while data of two groups were compared using unpaired *t* test. Data among multiple groups were compared via one‐way ANOVA and Tukey's post hoc test. Experiments were repeated 3 times

To investigate the upstream regulatory network of CXCL14 in the NK cell–related immunotherapy, we analysed transcription factors that could bind to CXCL14 promoter and enhancer by consulting an online database (https://regrna2.mbc.nctu.edu.tw/), which showed that CDX2 could bind both to the promoter and enhancer of CXCL14. Next, we analysed the expression of CDX2 in HNSCC patient samples by RT‐qPCR, which demonstrated that CDX2 expression was lower in HNSCC tissues than that in the adjacent normal tissues (Figure 4A), suggesting the potential significance of CDX2 in HNSCC. However, the expression of transcription factors of CXCL14 (Sp8 and Creb) in HNSCC tissues and adjacent normal tissues exhibited no significant difference (Figure S1A).

Subsequently, RT‐qPCR was applied to detect the expression of CDX2 in NK cells of peripheral blood of HNSCC patients and healthy volunteers. In comparison to NK cells of healthy volunteers, CDX2 expression was significantly decreased in NK cells of HNSCC patients (Figure 4B). Then, NK cells and NK‐92 cells were stimulated by IL‐2. CDX2 expression was up‐regulated in the IL‐2‐stimulated NK cells and NK‐92 cells compared with untreated controls (Figure 4C). Taken together, these data indicated the low expression of CDX2 in HNSCC and the derived NK cells.

We then proceeded to knock down CDX2 expression by lentivirus treatment in primary NK cells. Western blot analysis demonstrated that CDX2 expression was considerably decreased by sh‐CDX2‐1, sh‐CDX2‐2 and sh‐CDX2‐3 (Figure 4D), among which sh‐CDX2‐1 showed the best knockdown efficacy and was selected for the subsequent experiments. Meanwhile, CDX2 was overexpressed or silenced along with CXCL14 overexpression in primary NK cells. RT‐qPCR showed that CDX2 and CXCL14 expression was notably increased by oe‐CDX2, while the elevated CXCL14 expression by oe‐CXCL14 was suppressed by sh‐CDX2‐1 (Figure 4E). Furthermore, Western blot assay revealed no difference in CDK2/CXCL14 ratio among NK cells of different densities (Figure S1C). In summary, these results revealed that CDX2 positively regulated CXCL14 expression.

It has been reported that PAX‐6 and CDX2 interact with co‐activator p300 to synergistically activate glucagon gene expression.^20^ Thus, we speculated that CDX2 might regulate CXCL14 expression by interacting with the co‐activator p300. To test this prediction, we used an speculation, IP assay to determine the interaction between CDX2 and p300 in NK cells, which showed that CDX2 indeed interacted with p300 (Figure 4F). Next, to investigate the role of p300 in the regulation of CXCL14 by CDX2, we used lentivirus to knock down p300. Results demonstrated that p300 protein expression was significantly down‐regulated by sh‐p300‐1, sh‐p300‐2 and sh‐p300‐3 (Figure 4G). Meanwhile, we silenced p300 in the presence of CDX2 and conducted RT‐qPCR analysis, which revealed that CDX2 expression was not affected by sh‐p300, but that CXCL14 expression was significantly down‐regulated by sh‐p300 in the presence of CDX2 (Figure 4H).

Previous investigations demonstrated that CDX2 could bind to YAP1 enhancer to regulate its expression.^21^ P300, a histone acetyltransferase, could catalyse the acetylation of lysin27 on histone H3 (H3K27ac) in the promoter or enhancer regions.^22^, ^23^ Thus, we hypothesized that CDX2‐p300 complex might activate the enhancer through binding to CXCL14 enhancer. Since H3K27ac and H3K4me1 are enhancer markers, we applied ChIP assay to detect the enrichment of CDX2, H3K27ac, p300 and H3K4me1 in the enhancer region of CXCL14. Results demonstrated that the enrichment of CDX2, H3K27ac, p300 and H3K4me1 in the enhancer region of CXCL14 was notably increased upon overexpression of CDX2 (Figure 4I), whereas the expression of H3K27ac and H3K4me1 was not affected (Figure 4J). In the meantime, no significant difference was detected in the expression of H3K27ac and H3K4me1 in NK cells of peripheral blood from healthy donors and HNSCC patients (Figure S1B). Altogether, CDX2‐p300 complex up‐regulated CXCL14 expression through activating the enhancer of CXCL14.

### 3.5 CDX2 enhances NK cell migration and cytolytic activity via up‐regulating CXCL14 expression

**FIGURE 5:**
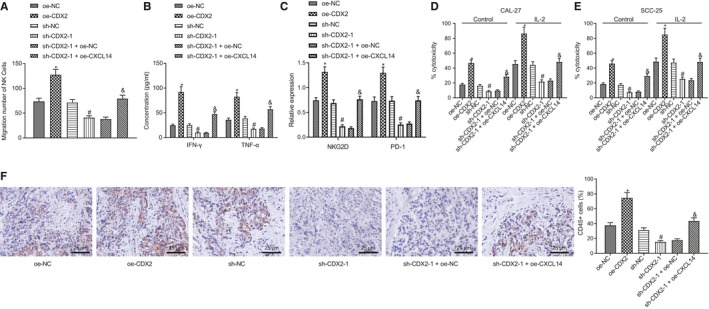
CDX2 enhances NK cell migration and cytolytic activity via up‐regulating CXCL14 expression. (A) Transwell assay to detect the effect of CXCL14 on the migration of NK cells. (B) ELISA to measure the levels of IFN‐γ and TNF‐α in the medium of NK cells. (C) Western blot assay to analyse the expression of NKG2D and PD‐1 in NK cells. (D) The cytotoxicity of NK cells to head and neck squamous cell carcinoma (HNSCC) cell line CAL27. (E) The cytotoxicity of NK cells to HNSCC cell line SCC‐25. (F) Immunohistochemistry to detect the expression of CD45 in SCC‐25 tumour spheroid. *P < .05 compared with oe‐NC, ^#^ *P* < .05 compared with sh‐NC group, and & *P* < .05 compared with sh‐CDX2 + oe‐NC group. Measurement data were presented as mean ± standard deviation. Data of two groups were compared using unpaired *t* test. Experiments were repeated 3 times

We next investigated the effects of CXCL14‐mediated CDX2 on the migration of NK cells and cytolytic activity. Our Transwell assay results revealed that NK cell migration was significantly increased by oe‐CDX2, but notably decreased by sh‐CDX2‐1. However, the NK cell migration suppressed by sh‐CDX2‐1 was substantially rescued by oe‐CXCL14 (Figure 5A). Then, ELISA results revealed that the expression of IFN‐γ and TNF‐α in the medium was significantly increased upon CDX2 overexpression, yet notably decreased by CDX2 knockdown, while the reduction in expression of IFN‐γ and TNF‐α caused by sh‐CDX2‐1 was reversed by oe‐CXCL14 (Figure 5B). Western blot results demonstrated that NKG2D expression and PD‐1 expression were remarkably increased after CDX2 overexpression, but considerably decreased after CDX2 silencing. The reduced NKG2D expression and PD‐1 expression induced by sh‐CDX2‐1 were rescued by oe‐CXCL14 (Figure 5C).

Next, we investigated the cytotoxic effect of IL‐2‐stimulated NK cells on HNSCC cell lines, CAL27 and SCC‐25. The cytotoxicity of IL‐2‐stimulated NK cells against HNSCC cells was significantly increased by oe‐CDX2, but notably decreased by sh‐CDX2‐1. However, the cytotoxicity of NK cells suppressed by sh‐CDX2‐1 was considerably enhanced by oe‐CXCL14 (Figure 5D,E).

Finally, we performed tumour spheroid assays using the SCC‐25 cell line in conjunction with immunohistochemistry. Results indicated that the number of infiltrated NK cells in SCC‐25 tumour spheroids was significantly increased after CDX2 overexpression, but was notably decreased after CDX2‐1 knockdown. The reduced number of infiltrated NK cells in SCC‐25 tumour spheroid caused by sh‐CDX2‐1 was rescued by oe‐CXCL14 treatment (Figure 5F). These data revealed that CDX2 accelerated NK cell migration and enhanced cytolytic activity via up‐regulating CXCL14 expression.

### 3.6 CDX2/CXCL14 inhibits in vivo tumorigenesis

**FIGURE 6:**
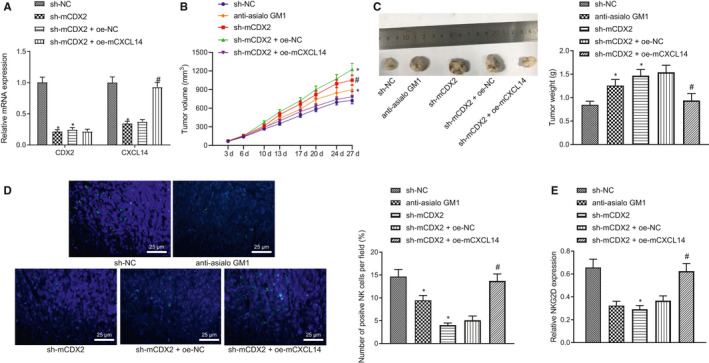
CDX2/CXCL14 inhibits the growth of head and neck squamous cell carcinoma (HNSCC) in vivo. (A) RT‐qPCR to detect the expression of CDX2 and CXCL14 in tumours. (B) The measurement of tumour volume. (C) The measurement of tumour weight. (D) IF to detect the NK cell infiltration in tumours. (E) Western blot assay to analyse the expression of NKG2D in mouse tumours. *P < .05 compared with sh‐NC group, and ^#^ *P* < .05 compared with sh‐mCDX2 + oe‐NC group. Tumours were excised 3 wks after lentivirus injection, and RT‐qPCR, IF and Western blot assay were performed. Measurement data were presented as mean ± standard deviation. Data among multiple groups were processed via one‐way ANOVA and Tukey's post hoc test, and tumour volume data from different time‐points were analysed by repeated measures ANOVA. Experiments were repeated 3 times

To investigate the role of CDX2/CXCL14 in HNSCC in vivo, we established the SCC7‐transplanted mouse model and injected CDX2 and/or CXCL14 overexpression or knockdown lentiviruses into the mice. First, RT‐qPCR data uncovered that the expression of CDX2 and CXCL14 was considerably down‐regulated in the sh‐mCDX2 group compared to that in the sh‐NC group. However, compared with sh‐mCDX2 + oe‐NC, CDX2 expression was unchanged, and CXCL14 expression was notably up‐regulated in the sh‐mCDX2 + oe‐mCXCL14 group (Figure 6A).

As expected, the tumour volume and weight were significantly increased when CDX2 was knocked down, both of which effects were remarkably decreased after restoration of CXCL14 (Figure 6B,C). Meanwhile, the data showed that the number of infiltrating CD45^+^ cells was remarkably decreased by CDX2 knockdown in vivo, which effect was counteracted after restoration of CXCL14 (Figure 6D). Consistent with the in vitro results, NKG2D protein expression was reduced by CDX2 knockdown, while CXCL14 overexpression reversed this reduction (Figure 6E). Taken together, our data revealed that CDX2/CXCL14 inhibited the growth of HNSCC in mice, which was associated with the function of NK cells.

## 4 DISCUSSION

Head and neck squamous cell carcinoma is a relative common cancer and one of leading causes of cancer death worldwide. NK cells serve as key players of innate immune system and exert important roles in the defence against tumours.^24^ In this paper, our primary goal was to elucidate the molecular mechanism by which NK cells exert an anti‐tumour action in HNSCC. Our data demonstrated that CXCL14 expression was decreased in HNSCC and NK cells, but was elevated in IL‐2‐simulated NK cells. Overexpression of CXCL14 could enhance the migration and cytotoxicity of NK cells in vitro. CXCL14 functions in a NK cell–dependent manner in vivo to suppress HNSCC development in mice. Our mechanism study reveals that CXCL14 expression is up‐regulated by CDX2 through activation of its enhancer. CDX2 can increase NK cell migration and cytolytic activity *via* up‐regulation of CXCL14, and the CDX2/CXCL14 axis inhibits the tumorigenesis of HNSCC in mice through NK cells.

CXCL14 has been implicated as a novel immune regulator to mediate inflammatory cell migration and thus facilitate their immunological functions against pathogens and tumours.^10^, ^25^, ^26^ Ozawa et al^17^ revealed that CXCL14 up‐regulation by gefitinib could be beneficial for HNSCC suppression in vivo. In their subsequent research, Knodo et al demonstrated that restoration of CXCL14 mRNA expression enhanced the anti‐tumour effect of cetuximab in HNSCC.^27^ We have now shown that CXCL14 expression was down‐regulated in HNSCC and patient NK cells, which suggests its role in the development of HNSCC and in the regulation of NK cells. Our gain‐ and loss‐of‐function studies uncovered that overexpression of CXCL14 enhanced NK cell migration and infiltration, up‐regulated the expression of IFN‐γ and TNF‐α and NKG2D, and increased NK cell cytotoxicity, which was in line with previous studies.

Natural killer cells are emerging as immunotherapy candidates in cancer due to their rapid and efficient immunosurveillance and mobilization to destroy tumour cells,^6^, ^28^ in which the cytolytic activity of NK cells can be exerted by expression of perforin and granzymes.^29^ Meanwhile, IFN‐γ and TNF‐α have been revealed to be involved in the clearance of virus and tumours.^30^, ^31^ Wang et al^32^ demonstrated that NK cells can induce targeted cytolysis by producing IFN‐γ and TNF‐α release. Our data suggested that NK cells responded to HNSCC by secreting IFN‐γ and TNF‐α, and also by increasing their infiltration through CXCL14 overexpression. Of note, our results also showed the up‐regulation of natural killer group 2D (NKG2D) by CXCL14 overexpression. NKG2D is a receptor that is expressed on the surface of NK cells as well as T cell subsets, which facilitates crucial immunosurveillance roles by recognizing ligands that are usually overexpressed by cancerous cells.^33^ It has been reported that NKG2D expression is correlated with NK cell infiltration,^34^, ^35^ which also supports our present result. The in vivo studies in the tumour‐bearing mouse model also showed that overexpression of CXCL14 can inhibit the tumour growth and increase NK cell infiltration.

Next, we ask whether there is any upstream molecule that regulates the expression of CLCX14. To answer this question, we conducted bioinformatics analysis, which predicted CDX2 as an upstream regulator of CLCX14. CDX2 belongs to homeobox transcription factor family, which was initially identified as an important transcription factor for the development of intestinal epithelium.^36^ In colorectal cancer, CDX2 expression was found to be down‐regulated.^37^ Present results show that CDX2 expression is also down‐regulated in HNSCC and NK cells, while overexpression of CDX2 could increase the expression of CXCL14. Molecular biological analysis further revealed that CDX2 interacts with histone acetyltransferase p300 and that CDX2/p300 activates the enhancer of CXCL14 to promote its expression. Our data thus uncover the potential molecular mechanism by which CXCL14 is regulated. Finally, we investigated whether CDX2 contributes to the anti‐tumour potential of NK cells. Our in vitro and in vivo data consistently indicate that CDX2 induces the migration and infiltration of NK cells, increases the secretion of IFN‐γ and TNF‐α by NK cells, enhances NK cell toxicity against HNSCC, and suppresses the tumour growth in mice.

**FIGURE 7:**
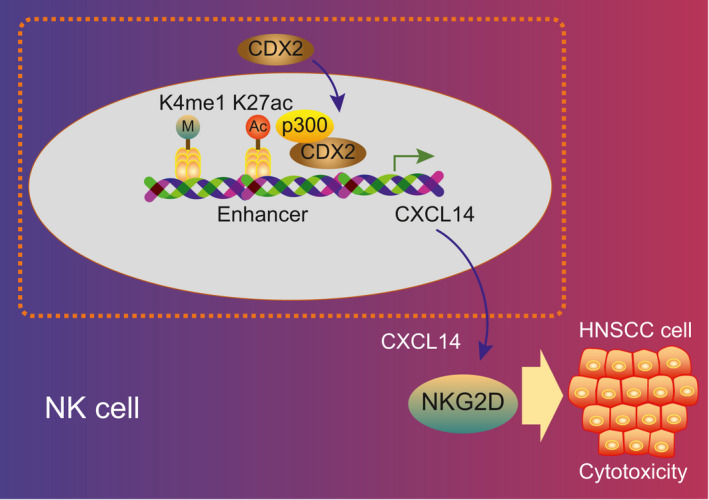
CDX2 promotes CXCL14 expression by activating its enhancer, which enhances NK cell–mediated immunotherapy against head and neck squamous cell carcinoma (HNSCC)

Taken together, our research elucidates the molecular mechanism how the CDX2/CXCL14 axis enhances NK cell–mediated immunotherapy against HNSCC, in which CDX2 stimulates the cytotoxicity of NK cells through up‐regulation of CXCL14 (Figure 7). Our study may also inform a novel immunotherapeutic strategy to be translated into the treatment of HNSCC. However, the possibility of detrimental immunotherapy side effects such as attack of healthy cells or tissues calls for careful consideration in future investigations.

## CONFLICT OF INTEREST

The authors declare that there is no conflict of interest.

## AUTHOR CONTRIBUTIONS

**Haitao Wang:** Methodology (equal); Writing‐original draft (equal); Writing‐review & editing (equal). **Shanji Nan:** Methodology (equal); Writing‐original draft (equal); Writing‐review & editing (equal). **Ying Wang:** Data curation (equal); Formal analysis (equal). **Chengbi Xu:** Resources (equal).

## Supporting information

Figure S1

## Data Availability

The data sets generated and/or analysed during the current study are available from the corresponding author on reasonable request.
